# Health-related quality of life in breast cancer patients in Asia: A meta-analysis and systematic review

**DOI:** 10.3389/fonc.2022.954179

**Published:** 2022-09-28

**Authors:** Xinyu Chen, Chenxi Wu, Dingxi Bai, Jing Gao, Chaoming Hou, Tingting Chen, Lulu Zhang, Huan Luo

**Affiliations:** School of Nursing, Chengdu University of Traditional Chinese Medicine (TCM), Chengdu, China

**Keywords:** Asia, breast cancer, health-related quality of life, meta-analysis, systematic review

## Abstract

**Objectives:**

The primary purposes of this meta-analysis and systematic review were to evaluate the health-related quality of life (HRQoL) of Asian breast cancer (BC) patients to understand their holistic HRQoL level and provide medical and nursing recommendations to improve and preserve their quality of life.

**Methods:**

A comprehensive literature search was conducted to find cross-sectional studies published in Chinese and English concerning HRQoL in BC patients from the inceptions of databases to 14 March 2022. The databases consulted were PubMed, Web of Science, Embase, Cochrane, PsyclNFO, CINAHL, and CNKI. Literature screening, data extraction, risk bias assessment, and data synthesis were independently carried out by two researchers. The Endnote X9 and Stata 15.0 software programs were used during the meta-analysis process.

**Results:**

Out of the 8,563 studies identified, 23 cross-sectional studies involving 3,839 Asian BC patients were included in this meta-analysis. Two tools, namely, European Organization for the Research and Treatment of Cancer Quality of Life Questionnaire C30 (EORTC QLQ-C30) and Quality of Life Questionnaire Breast Cancer module 23 (EORTC QLQ-BR23)—were used to evaluate the HRQoL of BC patients in Asia. The pooled mean of the global health status of Asian BC patients was 58.34 (95% confidence interval [CI]: 53.66–63.02). According to functional subscales of EORTC QLQ-C30 and EORTC QLQ-BR23, Asian BC patients suffered from the worst emotional functioning (pooled mean=66.38; 95% CI: 59.66–73.11) and sexual enjoyment (pooled mean=49.31; 95% CI: 31.97–63.36). In addition, fatigue (pooled mean=42.17; 95% CI: 34.46–49.88) and being upset by hair loss (pooled mean=48.38; 95% CI: 36.64–60.12) were the most obvious symptoms that Asian BC patients experienced according to the meta-analysis results of the EORTC QLQ-C30 and EORTC QLQ-BR23 symptom subscales.

**Conclusion:**

Asian BC patients experience a relatively low HRQoL due to the prominent decline in their body functions, as well as the unpleasant experiences caused by their symptoms. It is suggested that timely, appropriate, and targeted intervention should be provided in relation to the physical, psychological, and social aspects of Asian BC patients’ lives to enhance their ability to function, relieve them of adverse symptoms, and improve their overall HRQoL.

**Systematic Review Registration:**

https://www.crd.york.ac.uk/PROSPERO/, identifier CRD42022321165.

## Introduction

Breast cancer (BC), the most common malignant tumor, with the highest mortality among women globally, continues to threaten women’s lives, with an increasing number of cases yearly. The latest data pertaining to cancer worldwide in 2020 show that the number of new cases of BC has reached 2.26 million (11.7%), surpassing that of lung cancer, and so BC has become the world’s leading cancer (1, 2). Various factors may increase the likelihood of incidence of BC, and may also be influenced by socioeconomic developments, ethnicity, and lifestyle, among others (3–5). There are significant differences between Western countries and Asian countries in terms of clinical presentation, epidemiology, treatment methods, and prognosis of the disease. For instance, developing countries in Asia have a lower incidence of BC than Western countries but have a higher mortality rate (6). In addition, there are differences in the choice of BC treatment strategies between Asian and Western countries. To preserve their self-image and sexuality, young women with early-stage BC are more likely to choose breast-conserving surgery (BCS) as the primary treatment (7, 8). However, despite BC being diagnosed in Asian women at an earlier age approximately 10 years earlier than in Western countries, and more than 50% of Asian BC patients suffering from locally advanced cancers (9–11), findings from many studies have demonstrated that breast-conserving surgery is less common in Asia than in Europe and the United States (12, 13). The unique characteristics of BC in Asia and the different therapies adopted by Asian BC patients have led to a different health-related quality of life (HRQoL) compared to other continents.

HRQoL refers to an individual’s health status under the influence of illness and injury, medical intervention, aging, and changes in the social environment, as well as subjective satisfaction related to one’s economic and cultural backgrounds and values orientation (14). Owing to the complex treatment and prognosis of BC, patients usually experience chronic or prolonged diagnostic procedures and treatment processes, such as surgery, chemotherapy, radiotherapy, and hormonotherapy, which consequently lead to severe physical disorders, including breast removal, skin discoloration, hair loss, fatigue, sexual dysfunction, and distortions in body image (15, 16). However, as a chronic disease, BC usually affects more than just the body’s integrity (17). The treatment experiences and associated symptoms in BC patients also give rise to negative effects on mental health. Anxiety, depression, and distress in BC patients are found at high levels, even years after the acute phase or successful treatment in some cases (18–20). These negative effects on mental health can be caused by various factors, including, but not limited to, treatment-related side effects, interruption of the desire for childbearing (or even loss of fertility), and fear of cancer recurrence (FCR) (21, 22).

Impaired HRQoL is the most accurate indication of the gap between an individual’s actual functional level and the ideal standard (23). In Asia, a higher proportion of cancers are diagnosed at advanced stages due to a lack of early screening and insufficient understanding of the disease. Therefore, cancer patients in Asia tend to have more severe symptoms, such as pain, fatigue, insomnia, and anxiety, which are more likely to lower their HRQoL. There exists a consensus that the treatment of cancer patients should not only control their pathological reactions and relieve the discomfort caused by the disease and treatment but also reduce their psychological and emotional distress to improve their holistic HRQoL. However, in the context of the different types of economic and medical statuses, as well as cultural and habitual practices, such as the use of traditional medicine, there is a lack of overall understanding of the level of HRQoL of Asian BC patients (24, 25). Therefore, this meta-analysis aims to investigate the holistic HRQoL of Asian BC patients and provide conclusive evidence for developing more targeted measures to improve their quality of life.

## Materials and methods

This research was conducted according to the Preferred Reporting Items for Systematic Review and Meta-Analyses (PRISMA) guidelines (26). The current study’s protocol registration number in PROSPERO: International prospective register of systematic reviews is CRD42022321165.

### Search strategy

Six English databases (PubMed, Web of Science, Embase, Cochrane, Psyclnfo, and CINAHL) and one Chinese database (CNKI) were searched to retrieve eligible articles from the inceptions of databases to 14 March 2022. The search terms “breast cancer,” “breast neoplasm,” “breast tumor,” “health-related quality of life,” “quality of life,” “Asia,” “Far East,” “Southeast Asia,” “South eastern Asia,” “Asia, Western,” “Middle East,” “China,” “Chine*,” “Hong Kong*,” “Macau,” “Tibet*,” “Taiwan*,” “Japan*,” “Korea*,” “Mongoli*,” “India*,” “Brunei*,” “Indonesia*,” “Lao*,” “Malay*,” “Myanmar,” “Burmese*,” “Philippines*,” “Singapore*,” “Thai*,” “Timor*,” “Vietnam*,” “Bangladesh,” “Bengal*,” “Bhutan*,” “India*,” “Nepal*,” “Pakistan*,” “Sri Lanka*,” “Kazakhstan,” “Tajikistan,” “Turkmenistan,” and “Borneo” were used in various combinations to ensure the capture of related literature. The asterisk (*) symbol is significant as it is the component of the search strategy. **Appendix S1** in the **Supplementary Materials** reveals the search strategies used in the seven databases above. Furthermore, we checked the references listed in the selected articles for additional eligible resources.

### Inclusion and exclusion criteria

#### Inclusion criteria

•Populations: Asian adults (age ≥ 18 years old) diagnosed with breast cancer at any stage of pathology
•Study type: cross-sectional studies that investigated HRQoL of BC patients
•Outcomes: HRQoL scores of BC patients were evaluated by related tools

#### Exclusion criteria

•Patients with other diseases aside from breast cancer
•Patients who had cognitive impairment or any psychiatric disorder
•Studies not in English or Chinese, gray literature, and studies that are not original
•Incomplete information or studies without the full text available
•Unpublished data and presentations that did not provide data that were accurate and clear with respect to the research variables

### Study selection and data extraction

Two researchers (XYC and CXW) screened all of the literature by reading the titles and abstracts, then they excluded the studies that did not clearly meet the inclusion criteria; the researchers then read the full text to determine which should be used in our study. The whole process of screening and reading was carried out by the two researchers independently, and a third reviewer (TTC) stepped in when there was any discrepancy between the first two researchers. Once the literature was chosen, both researchers (XYC and CXW) independently extracted the information regarding the authors, region of the country, year of publication, study design, sampling methods and settings, HRQoL instruments, etc. Finally, all the information was integrated and verified by the two researchers.

### Quality appraisal

The quality of the eligible studies was assessed *via* the Joanna Briggs Institute tool for cross-sectional studies (JBI Critical Appraisal Checklist for Analytical Cross-Sectional Studies) (27). This tool includes eight aspects of every cross-sectional study, evaluated by “Yes,” “No,” and “Unclear.” The answers of “yes” ≥ 5, 3–4, and 0–2 times are considered to indicate high, moderate, and low methodological quality, respectively. Similarly, two researchers (XYC and CXW) conducted the quality evaluation procedure independently, and the third researcher (LLZ) was asked to address any divergence.

As recommended by the Cochrane Collaboration, we used the Risk of Bias in Non-Randomized Studies - of Intervention (ROBINS-I) tool to assess the risk of bias in the studies included (28) from the following seven domains: (i) bias in the selection of exposed and non-exposed cohorts; (ii) bias in the assessment of exposure; (iii) bias in the presence of outcome of interest at the start of study; (iv) bias in the control of prognostic variables (with matching or adjusting); (v) bias in the assessment of the presence or absence of prognostic factors; (vi) bias in the assessment of outcome; and (vii) bias in adequacy regarding follow-up of cohorts.

### Statistical analysis

Stata 15.0 software was used to conduct the meta-analysis, and the pooled mean value was taken as the effect size (ES). The pooled mean of Asian BC patients’ HRQoL was estimated using a random effects model with a confidence interval of 95%, and the scores were from the “global health status” for Quality of Life Questionnaire C30 (QLQ-C30), functional scales, and symptom scales for both QLQ-C30 and Quality of Life Questionnaire Breast Cancer module 23 QLQ-BR23. Forest plots were used to present the pooled means (95% CI) of the studies included. Statistical heterogeneity was assessed with the I^2^ statistic, and I^2^ values above 50% were interpreted as heterogeneous. Meta-regression was then used to analyze the factors related to high heterogeneity. Publication bias was assessed *via* Begg’s test, and a *p*-value greater than 0.05 (*p* > 0.05) indicated no publication bias. If publication bias existed, the trim-and-fill method was employed to detect the effects of publication bias on the results. To assess the influence of each study on the pooled ES, sensitivity analysis was conducted by omitting an individual study each time and repeating the analysis.

## Results

### Study selection and data characteristics

**Figure 1:**
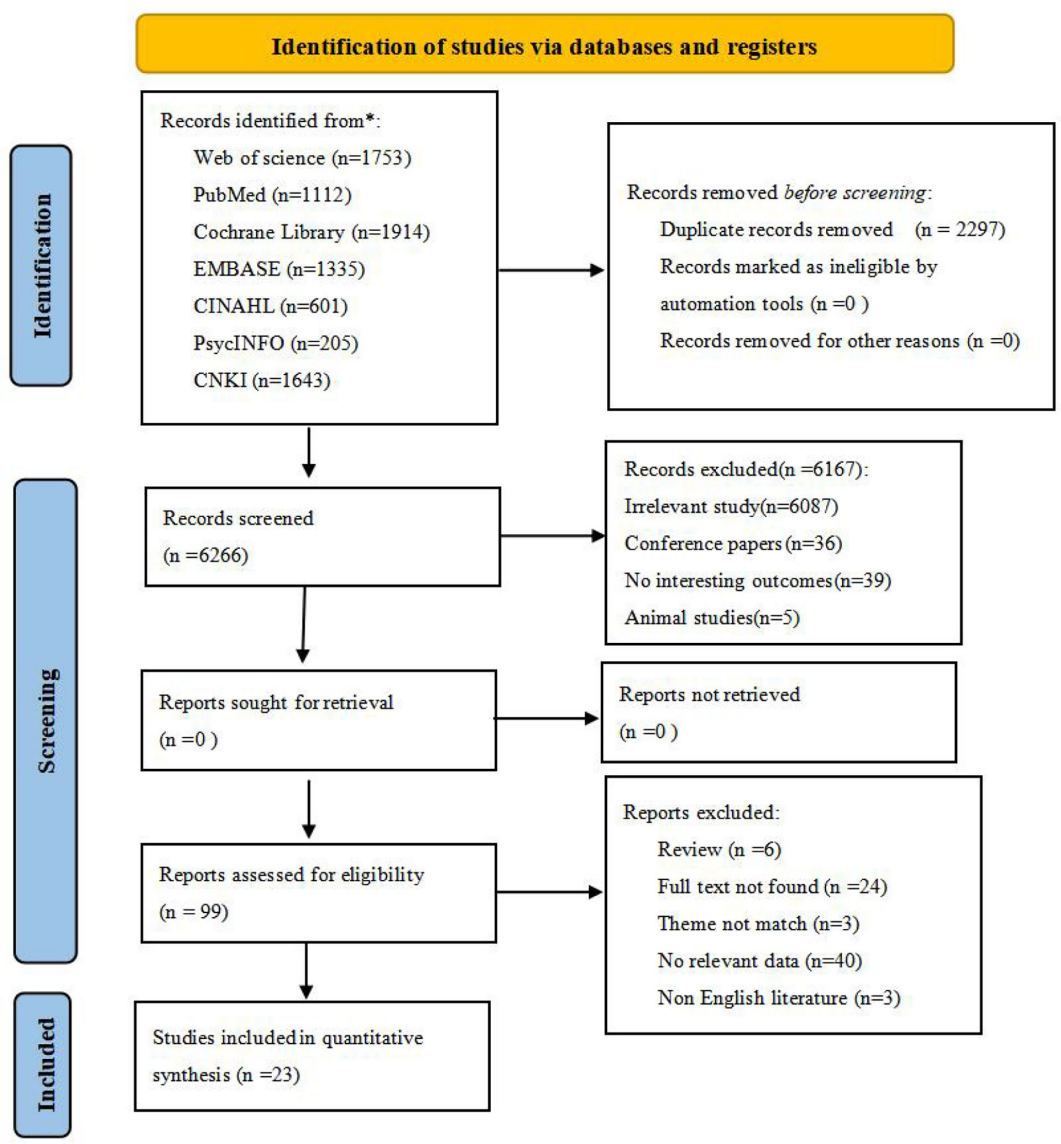
Flow diagram of the search strategy and study selection.From: Page MJ, McKenzie JE, Bossuyt PM, Boutron I, Hoffmann TC, Mulrow CD, et al. The PRISMA 2020 statement: an updated guideline for reporting systematic reviews. BMJ 2021;372:n71. doi: 10.1136/bmj.n71For more information, visit: http://www.prisma-statement.org/. * reporting the number of records identified from each database or register searched (rather than the total number across all databases/registers).

After screening and removing duplicates, 23 cross-sectional studies (29–51) containing 3,839 Asian BC patients were included in this study from 8,563 retrieved records. The flow diagram of literature screening is displayed in **Figure 1**.

### Quality appraisal

The characteristics of the 23 cross-sectional studies are given in **Table 1**. The details of quality assessment for the studies included are presented in **Appendix S2** in the **Supplementary Materials** section, which shows that the scores of the studies included range from 5 to 8, all being of high methodological quality. Moreover, the assessment results for the risk of bias are given in **Appendix S3** in the **Supplementary Materials**. There are 5 studies with a high risk of bias, 14 with a moderate risk of bias, and 4 with a low risk of bias.

**Table 1:** Characteristics of enrolled research.

Study ID, Year	Country	Study design	Sampling method	Setting	Mode of data collection	Instrument	Participants	Age (mean)
Dubashi, 2010 (29)	India	Cross-sectional study	Convenience	Breast clinic	Self-report	QLQ-C30 and QLQ-BR23	51	35
Ghufran, 2013 (30)	Bahrain	Cross-sectional study	Simple random sample	Hospital oncology center	Interview	QLQ-C30 and QLQ-BR23	337	50.2
Min, 2020 (31)	Myanmar	Cross-sectional study	Convenience	Cancer clinic	NR	QLQ-C30	74	–
Chen, 2018 (32)	China	Cross-sectional study	Consecutive	Oncology wards	Interview	QLQ-C30 and QLQ-BR23	608	48
Muna, 2018 (33)	Nepal	Cross-sectional study	Purposive sampling	Outpatient departments	NR	QLQ-C30 and QLQ-BR23	107	47.88
Huang, 2019 (34)	China	Cross-sectional study	Convenience	Breast Cancer Alliance	NR	QLQ-C30 and QLQ-BR23	193	55.52
Najaf, 2016 (35)	Iran	Cross-sectional study	Consecutive	Medical oncology clinic	Self-report	QLQ-C30	155	47.6
Fatemeh, 2021 (36)	Iran	Cross-sectional study	Convenience	Oncology centers	Interview	QLQ-C30 and QLQ-BR23	190	46.9
Safaee, 2008 (37)	Iran	Cross-sectional study	Consecutive	Chemotherapy ward	NR	QLQ-C30	119	48.27
Almutairi, 2016 (38)	Saudi Arabia	Cross-sectional study	Consecutive	Outpatient units	Self-report	QLQ-C30 and QLQ-BR23	145	–
Najmeh, 2013 (39)	Iran	Cross-sectional study	Convenience	Breast Cancer Research Center	NR	QLQ-C30 and QLQ-BR23	68	48
Aishwarya, 2019 (40)	India	Cross-sectional study	Convenience	Department of Oncology	Self-report	QLQ-C30 and QLQ-BR23	50	54.02
Ganesh, 2016 (41)	Malaysia	Cross-sectional study	Systematic random sampling	Oncology clinic	NR	QLQ-C30 and QLQ-BR23	223	52.4
Sajani, 2014 (42)	Nepal	Cross-sectional study	Convenience	National cancer centers	Interview	QLQ-C30 and QLQ-BR23	100	46.79
Azlina, 2013 (43)	Malaysia	Cross-sectional study	Consecutive	Public referral hospitals for breast cancer	NR	QLQ-C30 and QLQ-BR23	58	50.72
Ahmet, 2009 (44)	Turkey	Cross-sectional study	Consecutive	Department of Oncology	Interview	QLQ-C30	55	48.2
Huang, 2017 (45)	China	Cross-sectional study	Convenience	Medical centers	Interview	QLQ-C30	252	54.48
Syarifah, 2022 (46)	Malaysia	Cross-sectional study	Convenience	Oncology clinics	Interview	QLQ-C30	160	51.5
Huda, 2012 (47)	Lebanon	Cross-sectional study	Sequential sampling procedure	Medical Center	Interview	QLQ-C30	89	49.19
Shafika, 2009 (48)	Kuwait	Cross-sectional study	Consecutive	Medical oncology department	Interview	QLQ-C30 and QLQ-BR23	345	48.3
Fatemeh, 2017 (49)	Iran	Cross-sectional study	Convenience	Imam Reza Center	Interview	QLQ-C30	94	45.20
Saleha, 2010 (50)	Pakistan	Cross-sectional study	Consecutive	Department of Clinical Oncology	Interview	QLQ-C30 and QLQ-BR23	200	46.3
Fahimeh, 2018 (51)	Iran	Cross-sectional study	Convenience	Hospitals	Interview	QLQ-C30	166	50

Nothing: NR, no report.

### Outcomes of meta-analysis

#### Instruments

Two types of standard tools are used alone or in combination with each other for the studies included: the European Organization for the Research and Treatment of Cancer Quality of Life Questionnaire C30 (EORTC QLQ-C30) (n=23) and the Quality of Life Questionnaire Breast Cancer module 23 (QLQ-BR23) (n=13). QLQ-BR23 is a supplement for the general cancer questionnaire QLQ-C30, and it aims to identify unique concerns of the BC patients (41). The detailed analysis of Asian BC patients’ HRQoL, evaluated by the two tools, is as follows.

### Meta-analysis results based on the European Organization for the Research and Treatment of Cancer Quality of Life Questionnaire C30

**Figure 2:**
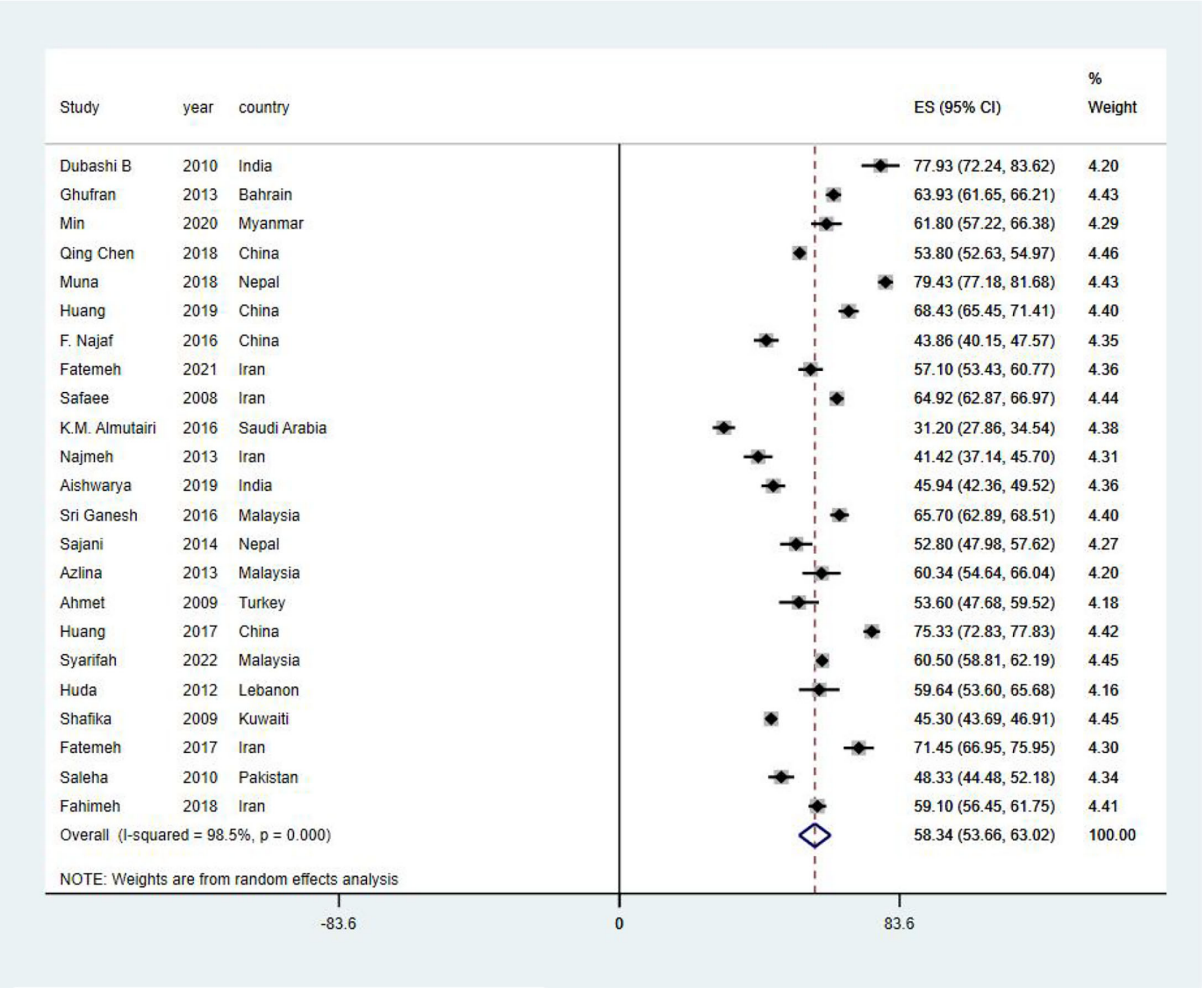
Global health status.

EORTC QLQ-C30 was used in all studies included in this systematic review to assess HRQoL of Asian BC patients. The mean value of the global health status of Asian BC patients ranged from 31.2 to 79.43, and the pooled mean value of global health status was 58.34 (95%CI: 53.66–63.02) (I^2 =^ 98.5%) by using the random effects model (**Figure 2**). Two subscales of QLQ-C30 also illustrate the HRQoL of BC patients with respect to their functions and symptoms. The functional subscales evaluate the functional status with respect to physical, role, emotional, cognitive, and social functioning. There exist 23 studies (29–51) that have reported the results of physical, role, emotional, and cognitive functioning status, while 22 (29–32, 34–51) have provided the results of social functioning. The pooled means of functional status from low to high are as follows: emotional functioning (pooled mean=66.38; 95% CI: 59.66–73.11), social functioning (pooled mean=71.26; 95% CI: 64.97–77.54), role functioning (pooled mean=72.70; 95% CI: 66.06–79.33), physical functioning (pooled mean=73.15; 95% CI: 66.84–79.46), and cognitive functioning (pooled mean=75.53; 95% CI: 70.58–80.49, **Table 2**). As for the symptom subscales, a total of nine symptoms are included. All 23 studies (29–51) reported the symptoms of fatigue, pain, insomnia, and appetite loss; 22 (30–51) reported nausea and vomiting, dyspnea, constipation, and diarrhea; and 21 (29–36, 38–48, 50, 51) reported financial difficulties. Among the nine symptoms, fatigue was the most common symptom suffered by Asian BC patients, with a pooled mean score of 42.17 (95% CI: 34.46–49.88), followed by financial difficulties (pooled mean=39.07; 95% CI: 39.07–49.07), insomnia (pooled mean=34.96; 95% CI: 26.93–42.99), pain (pooled mean=32.51; 95% CI:25.61–39.42), appetite loss (pooled mean=26.80; 95% CI: 18.50–35.10), dyspnea (pooled mean=24.46; 95% CI: 17.02–31.91), constipation (pooled mean=22.52; 95% CI: 15.24–29.81), nausea and vomiting (pooled mean=18.47; 95% CI: 14.26–22.67), and diarrhea (pooled mean=15.46; 95% CI: 8.60–22.33; **Table 3**).

**Table 2:** Functional subscales of EORTC QLQ-C30.

Study ID, Year	Country	Functional subscales				
		Physical functioningES (95% CI)	Role functioningES (95% CI)	Emotional functioningES (95% CI)	Cognitive functioningES (95% CI)	Social functioningES (95% CI)
Dubashi, 2010 (29)	India	86.39 (81.57, 91.21)	87.01 (80.79, 93.23)	82.11 (75.74, 88.48)	89.25 (83.81, 94.69)	87.70 (80.95, 94.45)
Ghufran, 2013 (30)	Bahrain	74.92 (72.60, 77.24)	68.84 (65.00, 72.68)	63.41 (59.84, 66.98)	73.38 (70.19, 76.57)	77.52 (74.29, 80.75)
Min, 2020 (31)	Myanmar	80.40 (76.94, 83.86)	66.40 (59.72, 73.08)	73.30 (68.52, 78.09)	83.60 (79.09, 88.11)	80.40 (75.43, 85.37)
Chen, 2018 (32)	China	75.50 (74.13, 76.87)	77.40 (75.37, 79.43)	74.20 (72.63, 75.77)	76.90 (75.35, 78.45)	69.90 (67.95, 71.86)
Muna, 2018 (33)	Nepal	90.21 (89.06, 91.36)	98.28 (97.32, 99.24)	93.06 (91.88, 94.24)	97.19 (96.00, 98.38)	—
Huang, 2019 (34)	China	87.85 (86.24, 89.46)	89.38 (86.93, 91.83)	80.00 (77.36, 82.64)	75.61 (72.89, 78.33)	82.45 (79.41, 85.49)
Najaf, 2016 (35)	China	68.51 (65.42, 71.60)	71.17 (66.91, 75.43)	42.90 (38.88, 46.92)	74.43 (70.77, 78.10)	55.74 (51.63, 59.85)
Fatemeh, 2021 (36)	Iran	71.70 (68.87, 74.53)	72.70 (68.96, 76.44)	54.00 (50.08, 57.92)	77.20 (73.96, 80.44)	67.10 (62.79, 71.41)
Safaee, 2008 (37)	Iran	57.31 (53.04, 61.58)	65.27 (59.00, 71.54)	56.26 (50.72, 61.80)	72.27 (67.33, 77.21)	69.61 (63.69, 75.53)
Almutairi, 2016 (38)	Saudi Arabia	62.90 (58.90, 66.90)	67.60 (62.85, 72.35)	83.30 (79.61, 87.00)	68.30 (63.86, 72.74)	65.00 (59.19, 70.81)
Najmeh, 2013 (39)	Iran	63.14 (58.240, 68.04)	63.93 (57.82, 70.04)	43.38 (38.18, 48.58)	54.41 (48.46, 60.36)	47.55 (41.44, 53.66)
Aishwarya, 2019 (40)	India	55.86 (54.99, 56.73)	35.74 (29.77, 41.71)	33.97 (24.66, 43.28)	65.24 (55.64, 74.84)	58.24 (54.73, 61.75)
Ganesh, 2016 (41)	Malaysia	81.70 (79.39, 84.01)	82.30 (78.99, 85.61)	78.50 (75.89, 81.11)	84.10 (81.74, 86.46)	81.60 (78.74, 84.46)
Sajani, 2014 (42)	Nepal	71.40 (68.05, 74.75)	78.50 (73.64, 83.36)	46.40 (39.66, 53.14)	59.30 (53.11, 65.49)	45.20 (38.99, 51.41)
Azlina, 2013 (43)	Malaysia	76.32 (69.76, 82.88)	67.24 (56.98, 77.50)	65.80 (58.90, 72.70)	84.77 (79.87, 89.68)	75.00 (67.00, 83.00)
Ahmet, 2009 (44)	Turkey	63.10 (56.68, 69.52)	68.20 (59.85, 76.55)	71.50 (65.24, 77.76)	78.30 (71.22, 85.38)	63.40 (55.13, 71.67)
Huang, 2017 (45)	China	91.19 (89.83, 92.55)	94.05 (92.40, 95.70)	84.82 (82.46, 87.18)	77.12 (74.72, 79.52)	86.18 (83.63, 88.73)
Syarifah, 2022 (46)	Malaysia	83.10 (81.42, 84.78)	77.80 (75.70, 79.90)	93.70 (92.03, 95.37)	81.80 (79.62, 83.98)	97.00 (95.56, 98.44)
Huda, 2012 (47)	Lebanon	79.10 (74.72, 83.48)	73.41 (67.09, 79.73)	65.92 (60.01, 71.84)	84.45 (79.85, 89.05)	60.29 (54.37, 66.21)
Shafika, 2009 (48)	Kuwait	52.60 (50.62, 54.58)	55.10 (52.84, 57.36)	60.30 (57.93, 62.68)	59.90 (57.38, 62.42)	61.20 (58.81, 63.60)
Fatemeh, 2017 (49)	Iran	91.35 (89.40, 93.31)	86.70 (82.98, 90.42)	78.55 (74.81, 82.29)	81.56 (78.09, 85.04)	89.18 (85.91, 92.45)
Saleha, 2010 (50)	Pakistan	56.40 (52.60, 60.20)	61.00 (55.20, 66.80)	46.16 (41.03, 51.29)	60.66 (56.76, 64.56)	77.33 (72.99, 81.68)
Fahimeh, 2018 (51)	Iran	60.60 (56.43, 64.77)	61.40 (57.89, 64.91)	51.40 (48.19, 54.61)	74.90 (71.28, 78.52)	68.10 (65.06, 71.14)
Random pool ES (95% CI)		73.15 (66.84, 79.46)	72.70 (66.06, 79.33)	66.38 (59.66, 73.11)	75.53 (70.58, 80.49)	71.26 (64.97, 77.54)

**Table 3:** Symptom subscales of EORTC QLQ-C30.

Study ID, Year	Country	Symptom subscales								
		FatigueES (95% CI)	Nausea and vomitingES (95% CI)	PainES (95% CI)	DyspneaES (95% CI)	InsomniaES (95% CI)	Appetite lossES (95% CI)	ConstipationES (95% CI)	DiarrheaES (95% CI)	Financial difficultiesES (95% CI)
Dubashi, 2010 (29)	India	18.10 (12.04, 24.17)	—	19.60 (12.29, 26.91)	—	8.49 (1.44, 15.54)	5.88 (1.13, 10.63)	—	—	40.50 (29.61, 51.39)
Ghufran, 2013 (30)	Bahrain	35.28 (32.01, 38.55)	10.29 (7.00, 13.58)	29.97 (26.64, 33.30)	20.22 (16.98, 23.46)	30.12 (25.93, 34.32)	13.38 (10.43, 16.33)	17.99 (14.72, 21.26)	6.83 (4.81, 8.85)	34.58 (30.07, 39.09)
Min, 2020 (31)	Myanmar	22.80 (18.70, 26.90)	4.70 (2.15, 7.25)	18.50 (12.71, 24.29)	11.30 (6.38, 16.22)	29.30 (22.31, 36.30)	15.30 (9.79, 20.81)	18.50 (13.01, 23.99)	0.90 (-0.33, 2.13)	57.70 (50.23, 65.17)
Chen, 2018 (32)	China	34.00 (32.56, 35.44)	19.00 (17.29, 20.71)	28.90 (27.32, 30.48)	17.20 (15.44, 18.97)	31.40 (29.46, 33.34)	24.10 (22.09, 26.11)	24.60 (22.50, 26.70)	10.40 (8.90, 11.90)	34.60 (32.32, 36.88)
Muna, 2018 (33)	Nepal	80.36 (77.24, 83.48)	0.46 (-0.06, 0.98)	7.47 (5.84, 9.11)	0.93 (-0.25, 2.11)	4.98 (2.72, 7.24)	6.54 (4.02, 9.06)	0.93 (-0.12, 1.98)	1.24 (0.04, 2.44)	0.93 (-0.12, 1.98)
Huang, 2019 (34)	China	28.39 (25.85, 30.93)	7.14 (5.23, 9.05)	20.02 (17.45, 22.59)	12.09 (9.49, 14.69)	34.75 (30.77, 38.73)	10.99 (8.52, 13.46)	18.34 (15.12, 21.57)	10.41 (7.88, 12.94)	19.50 (15.74, 23.26)
Najaf, 2016 (35)	China	52.77 (48.87, 56.67)	34.68 (30.29, 39.07)	42.34 (38.30, 46.38)	41.21 (36.08, 46.34)	47.29 (42.43, 52.15)	43.91 (39.09, 48.73)	20.49 (16.10, 24.88)	17.11 (12.98, 21.24)	64.18 (59.32, 69.04)
Fatemeh, 2021 (36)	Iran	45.00 (41.28, 48.73)	19.70 (15.98, 23.43)	43.30 (39.49, 47.11)	16.80 (13.13, 20.47)	35.60 (30.89, 40.31)	23.20 (18.52, 27.88)	25.80 (21.21, 30.39)	11.60 (8.42, 14.79)	55.40 (50.08, 60.72)
Safaee, 2008 (37)	Iran	41.74 (36.91, 46.58)	16.39 (11.29, 21.49)	33.19 (28.11, 38.27)	16.25 (11.39, 21.11)	43.70 (36.40, 51.00)	22.69 (16.17, 29.21)	14.85 (9.58, 20.12)	3.92 (0.98, 6.86)	—
Almutairi, 2016 (38)	Saudi Arabia	76.20 (72.47, 79.93)	68.90 (56.50, 81.30)	76.20 (72.29, 80.12)	80.00 (75.56, 84.44)	84.10 (79.95, 88.25)	80.90 (76.52, 85.28)	59.30 (54.25, 64.35)	41.20 (35.93, 46.47)	52.00 (45.60, 58.40)
Najmeh, 2013 (39)	Iran	56.54 (51.30, 61.78)	26.23 (20.06, 32.40)	45.59 (40.22, 50.96)	24.02 (16.67, 31.37)	46.57 (38.69, 54.45)	35.78 (29.17, 42.39)	28.92 (20.72, 37.12)	15.69 (9.81, 21.57)	66.67 (59.17, 74.17)
Aishwarya, 2019 (40)	India	64.64 (56.47, 72.81)	11.82 (7.52, 16.12)	73.50 (67.08, 79.92)	39.78 (29.35, 50.21)	56.50 (44.93, 68.08)	24.42 (17.15, 31.69)	11.22 (5.55, 16.89)	2.64 (0.16, 5.12)	35.64 (29.35, 41.94)
Ganesh, 2016 (41)	Malaysia	28.90 (26.29, 31.51)	11.70 (9.26, 14.14)	18.80 (16.14, 21.46)	10.01 (7.57, 12.45)	21.30 (17.74, 24.86)	18.98 (15.62, 22.34)	9.90 (7.08, 12.72)	7.70 (5.43, 9.97)	40.10 (35.95, 44.25)
Sajani, 2014 (42)	Nepal	37.10 (32.55, 41.65)	20.30 (15.60, 25.00)	39.80 (34.80, 44.80)	19.00 (13.26, 24.74)	40.70 (32.53, 48.87)	38.00 (30.51, 45.49)	17.70 (11.80, 23.60)	11.70 (6.51, 16.89)	67.70 (62.17, 73.23)
Azlina, 2013 (43)	Malaysia	29.69 (22.42, 36.96)	6.61 (2.60, 10.62)	25.29 (17.27, 33.31)	6.90 (2.43, 11.38)	28.16 (18.96, 37.36)	22.99 (14.77, 31.22)	19.54 (13.11, 25.97)	4.60 (1.21, 7.99)	28.16 (18.68, 37.64)
Ahmet, 2009 (44)	Turkey	49.30 (42.80, 55.80)	24.80 (17.03, 32.57)	38.10 (30.30, 45.90)	19.20 (10.64, 27.76)	38.50 (29.62, 47.38)	33.00 (24.25, 41.75)	24.00 (16.18, 31.82)	16.20 (9.28, 23.12)	28.20 (20.03, 36.37)
Huang, 2017 (45)	China	19.27 (17.08, 21.46)	3.84 (2.58, 5.10)	11.57 (9.36, 13.78)	8.20 (6.21, 10.19)	26.06 (23.13, 28.99)	6.22 (4.14, 8.30)	15.08 (12.30, 17.86)	5.82 (4.13, 7.51)	13.89 (11.01, 16.77)
Syarifah, 2022 (46)	Malaysia	20.80 (19.04, 22.56)	0.20 (-0.21, 0.61)	14.20 (12.23, 16.17)	2.10 (0.60, 3.60)	7.50 (4.73, 10.28)	0.60 (-0.10, 1.30)	0.80 (-0.01, 1.61)	0.60 (-0.10, 1.30)	11.00 (8.36, 13.64)
Huda, 2012 (47)	Lebanon	34.58 (28.70, 40.46)	9.50 (5.78, 13.22)	32.40 (25.22, 39.58)	7.86 (3.81, 11.91)	33.71 (25.33, 42.10)	24.34 (16.27, 32.41)	10.86 (5.56, 16.16)	9.74 (4.40, 15.08)	37.45 (29.18, 45.73)
Shafika, 2009 (48)	Kuwait	38.90 (36.65, 41.15)	30.20 (27.62, 32.79)	43.80 (41.51, 46.09)	42.40 (39.47, 45.33)	42.70 (39.72, 45.68)	37.50 (34.55, 40.46)	27.80 (24.82, 30.78)	22.10 (19.20, 25.00)	31.20 (28.54, 33.84)
Fatemeh, 2017 (49)	Iran	14.42 (10.42, 18.43)	5.67 (3.03, 8.31)	12.41 (9.33, 15.49)	8.16 (4.93, 11.39)	14.89 (10.10, 19.49)	7.80 (3.80, 11.81)	10.28 (6.20, 14.36)	2.84 (0.72, 4.97)	—
Saleha, 2010 (50)	Pakistan	73.55 (70.02, 77.08)	28.00 (24.50, 31.50)	51.00 (46.23, 55.77)	62.67 (56.50, 68.84)	34.67 (29.65, 39.69)	42.67 (36.77, 48.58)	35.00 (30.33, 39.67)	45.33 (3.17, 47.49)	50.00 (44.38, 55.62)
Fahimeh, 2018 (51)	Iran	68.10 (65.06, 71.14)	63.20 (59.67, 66.73)	22.90 (14.44, 31.36)	74.70 (70.03, 79.37)	65.10 (60.72, 69.48)	78.50 (74.32, 82.68)	84.30 (80.62, 87.98)	92.40 (89.46, 95.34)	52.40 (48.73, 56.07)
Random pool ES (95% CI)		42.17 (34.46, 49.88)	18.47 (14.26, 22.67)	32.51 (25.61, 39.42)	24.46 (17.02, 31.91)	34.96 (26.93, 42.99)	26.80 (18.50, 35.10)	22.52 (15.24, 29.81)	15.46 (8.60, 22.33)	39.07 (29.06, 49.07)

### Meta-analysis results based on the European Organization for the Research and Treatment of Cancer Quality of Life Questionnaire Cancer module 23

There are 13 studies (29, 30, 32–34, 36, 38–40, 42, 43, 48, 50) that have used QLQ-BR23. The details of the results are displayed in **Table 4**. The QLQ-BR23 tool also has two subscales to assess the functions and symptoms of BC patients specifically. As can be seen in **Table 4**, all 13 studies reported the evaluated results of body image, future perspective, breast symptoms, and arm symptoms; and 12 reported the results of sexual functioning (29, 30, 32–34, 36, 38, 39, 42, 43, 48, 50), sexual enjoyment (29, 30, 32–34, 36, 38, 39, 42, 43, 48, 50), systemic side effects (29, 30, 32–34, 36, 38–40, 42, 48, 50), and upset by hair loss (30, 32–34, 36, 38–40, 42, 43, 48, 50). The pooled scores in the functional subscales ranged from 49 to 66: sexual enjoyment (pooled mean=49.31; 95% CI: 31.97–63.36), sexual functioning (pooled mean=50.77; 95% CI: 28.00–73.54), future perspective (pooled mean=53.81; 95% CI: 44.26–63.81), and body image (pooled mean=66.15; 95% CI: 61.08–71.21). Additionally, the pooled scores of the symptom subscale ranged from 29 to 49: breast symptoms (pooled mean=29.26; 95% CI: 20.12–38.40), arm symptoms (pooled mean=34.02; 95% CI: 24.91–43.13), systemic side effects (pooled mean=35.88; 95% CI: 25.76–46.00), and upset by hair loss (pooled mean=48.38; 95% CI: 36.64–60.12). The pooled results suggest that sexual enjoyment and being upset by hair loss are the most frequent problems faced by Asian BC patients.

**Table 4:** Functional and symptom subscales of EORTC QLQ-BR23.

Study ID, Year	Country	Functional subscales				Symptom subscales			
		Body imageES (95% CI)	Sexual functioningES (95% CI)	Sexual enjoymentES (95% CI)	Future perspectiveES (95% CI)	Systemic side effectsES (95% CI)	Breast symptomsES (95% CI)	Arm symptomsES (95% CI)	Upset by hair lossES (95% CI)	
Dubashi, 2010 (29)	India	80.44 (72.58, 88.30)	61.54 (51.34, 71.74)	58.15 (47.49, 68.81)	72.62 (63.34, 81.90)	13.04 (9.77, 16.31)	8.98 (4.10, 12.96)	15.52 (9.94, 21.11)	—	
Ghufran, 2013 (30)	Bahrain	75.64 (72.45, 78.83)	25.92 (22.74, 29.10)	48.56 (45.13, 51.99)	61.29 (57.09, 65.49)	19.27 (17.37, 21.17)	13.66 (11.73, 15.5)	36.58 (33.19, 39.97)	46.33 (41.75, 50.91)	
Chen, 2018 (32)	China	64.90 (62.91, 66.89)	89.00 (87.74, 90.26)	88.30 (86.74, 89.86)	51.50 (49.00.54.00)	24.70 (23.36, 26.04)	17.10 (15.53, 18.67)	20.20 (18.64, 21.76)	38.60 (36.19, 41.01)	
Muna, 2018 (33)	Nepal	74.62 (71.71, 77.53)	2.95 (1.17, 4.73)	27.77 (25.31, 30.23)	80.36 (77.24, 83.48)	7.07 (5.95, 8.19)	12.69 (11.44, 13.94)	10.87 (9.34, 12.41)	12.66 (9.31, 16.01)	
Huang, 2019 (34)	China	78.73 (75.51, 81.96)	15.51 (12.98, 18.04)	28.63 (24.92, 32.34)	57.64 (53.64, 61.64)	23.75 (21.25, 26.25)	18.30 (16.06, 20.55)	23.15 (20.25, 26.05)	34.46 (29.82, 39.10)	
Fatemeh, 2021 (36)	Iran	59.70 (54.58, 64.82)	20.60 (17.61, 23.59)	19.70 (16.47, 22.93)	40.50 (5.65, 45.35)	38.90 (34.45, 43.35)	1.60 (1.50, 1.70)	19.80 (16.62, 22.99)	57.70 (52.55, 62.85)	
Almutairi, 2016 (38)	Saudi Arabia	64.70 (58.89, 70.51)	52.30 (48.44, 56.16)	22.50 (17.99, 27.01)	76.30 (70.55, 82.05)	64.40 (59.91, 68.89)	65.10 (60.85, 69.35)	62.90 (58.98, 66.82)	64.40 (59.03, 69.77)	
Najmeh, 2013 (39)	Iran	43.63 (37.34, 49.92)	14.46 (10.72, 18.20)	14.71 (7.04, 22.38)	57.84 (49.45, 66.24)	46.01 (40.60, 51.42)	27.57 (22.67, 32.47)	41.18 (34.97, 47.39)	60.29 (52.28, 68.30)	
Aishwarya, 2019 (40)	India	53.12 (49.94, 56.31)	—	—	32.40 (24.78, 40.02)	60.78 (56.17, 65.40)	45.36 (40.16, 50.56)	49.50 (45.88, 53.12)	76.90 (72.13, 81.67)	
Sajani, 2014 (42)	Nepal	56.00 (48.28, 63.72)	87.70 (84.23, 91.17)	79.30 (74.15, 84.46)	43.30 (36.07, 50.53)	37.60 (33.58, 41.62)	35.60 (31.29, 39.91)	37.40 (32.19, 42.61)	40.30 (31.83, 48.77)	
Azlina, 2013 (43)	Malaysia	75.57 (68.74, 82.40)	77.30 (70.69, 83.91)	50.00 (44.52, 55.48)	44.25 (36.48, 52.02)	—	27.16 (21.46, 32.86)	21.84 (15.17, 28.51)	21.21 (15.43, 26.99)	
Shafika, 2009 (48)	Kuwait	61.80 (59.34, 64.26)	69.90 (67.41, 72.39)	61.50 (59.07, 63.93)	59.50 (56.13, 62.87)	40.10 (38.25, 41.95)	35.60 (32.92, 38.28)	38.20 (35.73, 40.67)	44.80 (41.68, 47.92)	
Saleha, 2010 (50)	Pakistan	70.5 (66.15, 74.86)	92.33 (89.52, 95.14)	92.33 (89.53, 95.13)	22.00 (18.08, 25.92)	55.90 (53.48, 58.32)	73.00 (68.69, 77.31)	65.33 (60.98, 69.68)	83.33 (78.46, 88.20)	
Random pool ES (95% CI)		66.15 (61.08, 71.21)	50.77 (28.00, 73.54)	49.31 (31.97, 63.36)	53.81 (44.26, 63.81)	35.88 (25.76, 46.00)	29.26(20.12, 38.40)	34.02 (24.91, 43.13)	48.38 (36.64, 60.12)	

### Heterogeneity, publication bias, and sensitivity analysis

**Figure 3:**
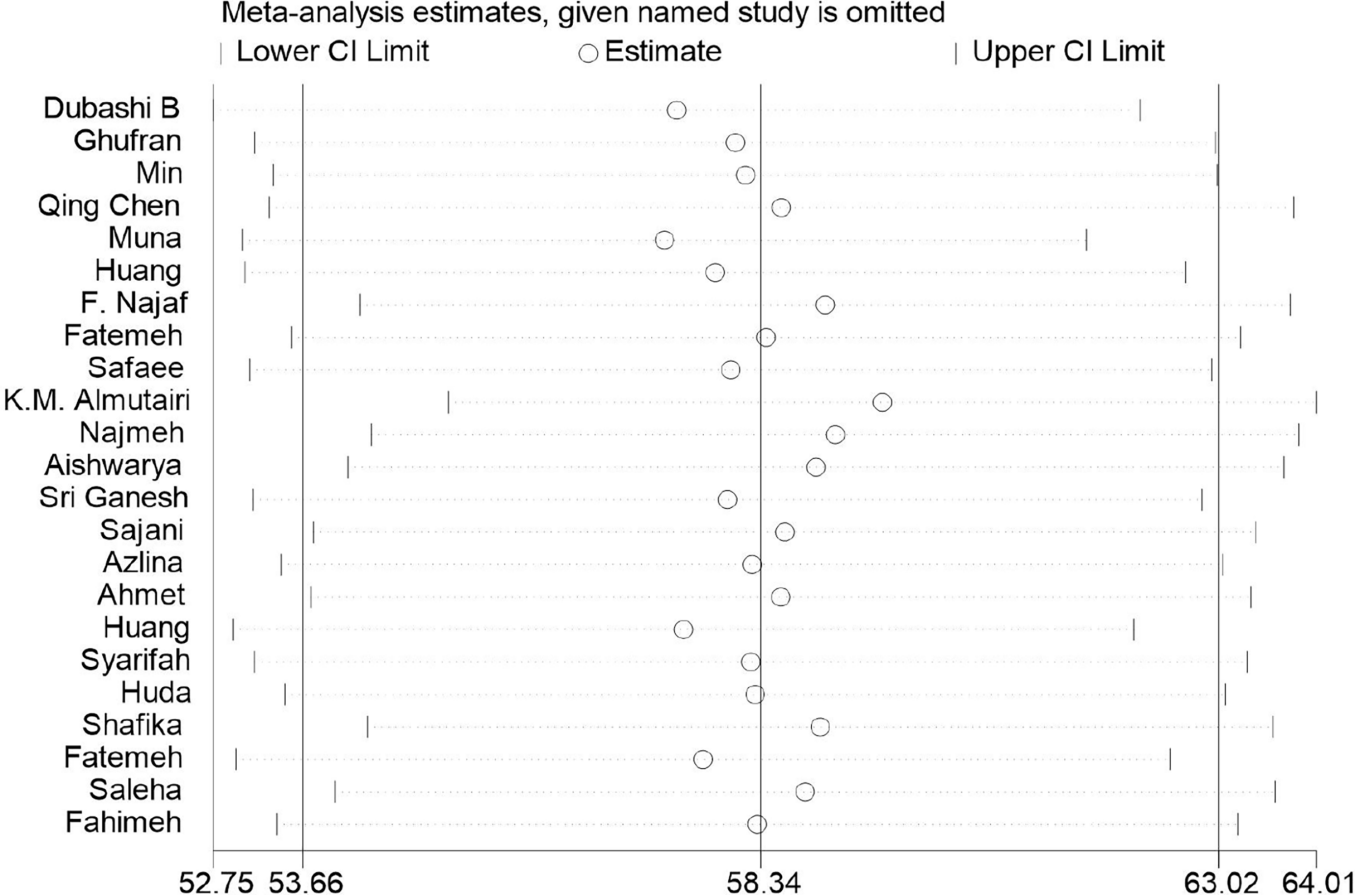
Results of sensitivity analysis plot of the global health status.

As illustrated in **Table 5**, the heterogeneity between the studies included was high, with all I^2^ values above 90%. According to the results of Begg’s tests, there was no obvious publication bias for the meta-analysis, except for the results for nausea and vomiting (*p*=0.048), dyspnea (*p*=0.032), and diarrhea (*p*=0.011) in the QLQ-C30 tool (**Table 5**). Moreover, the outcomes of the trim-and-fill method showed that the publication bias may have exerted an influence on the results for nausea and vomiting (*p*=0.914), dyspnea (*p*=0.057), and diarrhea (*p*=0.361, **Table 5**). Moreover, the sensitivity-analysis results indicated that no single study essentially changed the pooled mean of all the outcomes, and we take the sensitivity analysis plot of global health status as an example (**Figure 3**).

**Table 5:** Results of the publication bias and trim-and-fill method.

Tool		HRQoL	I*^2^* (%)	Studies included	Begg’s test		Trim-and-fill method	
					z	p	z	p
QLQ-C30	
	Functional subscales	Global health status	98.5	23	0.21	0.833	–	–
		Physical functioning	99.5	23	1.74	0.081	–	–
		Role functioning	99.1	23	0.11	0.916	–	–
		Emotional functioning	99.2	23	1.58	0.113	–	–
		Cognitive functioning	98.4	23	0.11	0.916	–	–
		Social functioning	98.7	22	0.73	0.463	–	–
	Symptom subscales	Fatigue	99.3	23	1.06	0.291	–	–
		Nausea and vomiting	99.3	22	1.97	0.048	1.298	0.194
		Pain	99.1	23	0.90	0.369	–	–
		Dyspnea	99.3	22	2.14	0.032	1.905	0.057
		Insomnia	98.9	23	0.85	0.398	–	–
		Appetite loss	99.4	23	0.95	0.342	–	–
		Constipation	99.4	22	0.23	0.822	–	–
		Diarrhea	99.6	22	2.54	0.011	0.914	0.361
		Financial difficulties	99.4	21	0.03	0.976	–	–
QLQ-BR23	Functional subscales	Body image	95.8	13	0.06	0.951	–	–
		Sexual functioning	99.9	12	0.21	0.837	–	–
		Sexual enjoyment	99.7	12	0.07	0.945	–	–
		Future perspective	98.3	13	0.18	0.855	–	–
	Symptom subscales	Systemic side effects	99.6	12	1.17	0.244	–	–
		Breast symptoms	99.7	13	0.31	0.760	–	–
		Arm symptoms	99.2	13	1.16	0.246	–	–
		Upset by hair loss	98.9	12	0.75	0.451	–	–

Begg's test: z: statistic; p: p value, the statistic significance of Begg's test. p<0.05 indicates than there is publication bias.Trim-and-fill method: z: statistic; p: p value, the statistic significance of the pooled results after trim-and- fill method.

### Meta-regression results based on the European Organization for the Research and Treatment of Cancer Quality of Life Questionnaire C30 and Quality of Life Questionnaire Breast Cancer module 23

We took the region of the country (in East Asia, Southeast Asia, South Asia, and in West Asia), the publication year (in 2008–2012, 2013–2017, and 2018–2022), the mean age of patients (age < 50; age ≥ 50), the sample size (1–200, 201–400, and 401–608), and the medical level worldwide (ranked below 50, ranked between 50 and 100,; and ranked above 100) (52) as covariates in the meta-regression. The results demonstrated that the five factors above could not explain the heterogeneity between studies of almost all the dimensions of HRQoL significantly through the univariate meta-regression method (*p*-value > 0.05), except the physical functioning dimension of the QLQ-C30, in which its high heterogeneity may be associated with the regions of countries (*p*=0.02, **Table 6** **)**.

**Table 6:** Meta-regression results.

Tool		HRQoL	Meta-regression (covariates)						
			Region of the country[*p*, Coefficient (95% CI)]	Publication year[*p*, Coefficient (95% CI)]		Mean age[*p*, Coefficient (95% CI)]	Sample size[*p*, Coefficient (95% CI)]		Medical level[P, Coefficient (95% CI)]
QLQ-C30		Global health status		[0.27, -2.49 (-7.01, 2.04)]	[0.66, 1.49 (-5.47, 8.46)]	[0.35, 4.82 (-5.70, 15.34)]		[0.60, -2.36 (-12.62, 6.90]	[0.65, 1.65 (-5.83, 9.12)]
	Functional scales	Physical functioning		[0.02, -5.20 (-9.32, -1.07)]	[0.17, 4.67 (-2.13, 11.46)]	[0.38, 5.14 (-6.88, 17.16)]		[0.90, -0.58 (-10.06, 8.90)]	[0.75, -1.20 (-8.79, 6.39)]
		Role functioning		[0.06, -4.63 (-9.46, 0.20)]	[0.63, 1.87 (-6.02, 9.77)]	[0.83, -1.48 (-15.35, 12.39)]		[0.58, 2.85 (-7.64, 13.35)]	[0.51, -2.73 (-11.14, 5.69)]
		Emotional functioning		[0.16, -4.37 (-10.55, 1.81)]	[0.52, 3.02 (-6.64.12.68)]	[0.42, 6.55 (-9.90, 23.00)]		[0.65, 2.91 (-10.03, 15.85)]	[0.61, -2.60 (-13.01, 7.82)]
		Cognitive functioning		[0.17, -2.61 (-6.38, 1.16)]	[0.31, 2.94 (-2.88, 8.76)]	[0.55, 2.94 (-7.24, 13.12)]		[0.62, -1.93 (-9.81, 5.94)]	[0.79, 0.82 (-5.57, 7.22)]
		Social functioning		[0.17, -3.37 (-8.34, 1.60)]	[0.51, 2.55 (-5.45, 10.55)]	[0.06, 12.09 (-0.39, 24.57)]		[0.94, 0.36 (-10.23, 10.96)]	[0.93, 0.36 (-8.39, 9.11)]
	Symptom scales	Fatigue		[0.06, 6.62 (-0.30, 13.55)]	[0.77, 1.63 (-9.68, 12.94)]	[0.38, -7.62 (-25.38, 10.13)]		[0.60, -0.02 (-0.08.0.05)]	[0.20, 7.41 (-4.27, 19.09)]
		Nausea and vomiting		[0.10, 5.32 (-1.03, 11.66)]	[0.54, -3.18 (-13.78, 7.42)]	[0.46, -5.21 (-19.53, 9.12)]		[0.59, 3.59 (-10.18.17.35)]	[0.74, -1.83 (-13.25, 9.59)]
		Pain		[0.07, 5.81 (-0.59, 12.20)]	[0.42, -4.04 (-14.27, 6.19)]	[0.36, -6.79 (-21.80, 8.21)]		[0.72, 2.45 (-11.35.16.25)]	[0.64, 2.55 (-8.53, 13.62)]
		Dyspnea		[0.10, 6.77 (-1.37, 14.90)]	[0.53, -3.84 (-17.44, 9.77)]	[0.89, -1.36 (-21.00, 18.23)]		[0.86, 1.53 (-16.20, 19.26)]	[0.72, 2.60 (-12.07, 17.26)]
		Insomnia		[0.18, 4.51 (-2.22, 11.24)]	[0.90, -0.66 (-11.24, 9.91)]	[0.90, 0.92 (-14.17, 16.00)]		[0.40.5.67 (-8.17, 19.50)]	[0.51, -3.61 (-14.84, 7.61)]
		Appetite loss		[0.09, 6.43 (-1.00, 13.86)]	[0.65, -2.64 (-14.56, 9.27)]	[0.58, -4.57 (-21.76, 12.63)]		[0.86, 1.39 (-14.57, 17.35)]	[0.85, -1.20 (-14.04, 11.63)]
		Constipation		[0.13, 5.04 (-1.66, 11.75)]	[0.95, 0.33 (-10.82, 11.48)]	[0.80, 2.08 (15.07, 19.22)]		[0.84, 1.42 (-12.99, 15.83)]	[0.86, -1.05 (-12.98, 10.89)]
		Diarrhea		[0.12, 5.89 (-1.62, 13.40)]	[0.86, -1.10 (-13.64, 11.43)]	[0.81, 2.37 (-18.09, 22.83)]		[0.69, -3.09 (-19.27, 13.08)]	[0.98, 0.15 (-13.28, 13.59)]
		Financial difficulties		[0.22, 4.36 (-2.85, 11.57)]	[0.57, -3.13 (-14.39, 8.13)]	[0.12, -13.88 (-31.62, 3.85)]		[0.83, -1.54 (-16.43, 13.35)]	[0.44, 4.27 (-7.17, 15.70)]
QLQ-BR23	Functional scales	Body image		[0.24 -3.43 (-9.48, 2.63)]	[0.70, -1.62 (-10.63, 7.39)]	[0.37, 6.68 (-9.01, 22.38)]		[0.92, 0.56 (-10.85, 11.96)]	[0.91, -0.45, (-9.51, 8.60)]
		Sexual functioning		[0.66, -4.12 (-24.00, 15.78)]	[0.09, -21.22 (-46.73, 4.29)]	[0.55, 15.32 (-70.40, 39.77)]		[0.35, 15.20 (-19.09, 49.48)]	[0.84, 2.75 (-26.73, 32.23)]
		Sexual enjoyment		[0.51, -4.93 (-21.07, 11.21)]	[0.19, -14.03 (-36.22, 8.15)]	[0.51, -12.90 (-55.78, 29.98)]		[0.13, 19.21 (-6.97, 45.39)]	[0.70, 4.24 (-19.85, 28.34)]
		Future perspective		[0.10, 0.03 (-10.17, 10.22)]	[0.96, -0.34 (-13.90, 14.58)]	[0.70, -4.27 (-27.79, 19.26)]		[0.82, 1.78 (-16.17, 19.74)]	[0.61, -3.34 (-17.39, 10.71)]
	Symptom scales	Systemic side effects		[0.18, 6.99 (-3.78, 17.75)]	[0.64, -3.41 (-19.04, 12.21)]	[0.90, 1.62 (-26.25, 29.48)]		[0.42, -7.47 (-27.02, 12.08)]	[0.80, 1.80 (-13.96, 17.56)]
		Breast symptoms		[0.30, 5.95 (-6.16, 18.06)]	[0.18, -10.60 (-26.88, 5.69)]	[0.97, -0.41 (-28.12, 27.30)]		[0.49, -7.09 (-29.10, 14.9)]	[0.46, 5.94 (-11.36, 23.24)]
		Arm symptoms		[0.07, 8.10 (-0.82, 17.02)]	[0.20, -8.35 (-21.74, 5.05)]	[0.86, 1.84 (-20.88, 24.57)]		[0.60, -4.41 (-22.57, 13.74)]	[0.57, 3.77 (-10.51, 18.05)]
		Upset by hair loss		[0.08, 9.42 (-1.53, 20.36)]	[0.34, -8.50 (-27.31, 10.30)]	[0.82, 3.43 (-35.54, 28.69)]		[0.60, -5.42 (-27.92, 17.08)]	[0.42, 6.80 (-11.36, 24.96)]

## Discussion

### The overall health-related quality of life of Asian breast cancer patients

This study shows that Asian BC patients have a global health status score of 58.34, similar to the results of research conducted by Hashemi (53) in the Middle East. However, compared with BC patients studied from other regions, Asian BC patients have a lower overall quality of life, especially when compared to those in Spain (54) or Germany (55). The literature review revealed that global health status is associated with many factors, such as the operation method (mastectomy or breast preservation) (29), presence of metastases (30), chemotherapy (56), and radiotherapy (57). In addition, the more comorbidities that BC patients have, the lower their quality of life. The survey conducted by Fu et al. (58) revealed that 20%–30% of BC patients present with comorbidities that were pre-existing or that developed after BC diagnosis, such as hypertension, arthritis, and diabetes (59). The research conducted by Miller et al., which addressed BC survivors in African-American and Latina groups, reached the same conclusion, i.e., that having fewer comorbidities means a better HRQoL (60). Therefore, it is necessary to help Asian BC patients to manage and control their comorbidities to enhance their HRQoL.

### The functional status of Asian breast cancer patients

QLQ-C30 and QLQ-BR23 evaluate the functional status of BC patients from different perspectives. The meta-analysis demonstrated that for Asian BC patients, emotional functioning and sexual enjoyment were the most severely impaired elements of quality of life during the progression of BC. In terms of emotional functioning, Asian BC patients’ scores were lower than those of Brazilian (61) and Mexican patients (62). This may be attributed to the culture of collectivism in Asia, which encourages the suppression of emotions to preserve interpersonal harmony (63). With the understanding that “sharing personal problems with others are [*sic*] regarded as unacceptable since it may make inappropriate demands on the group,” Asian BC patients tend to restrain their emotional disclosure (64). This suppression of emotions, however, leads to more negative moods on the one hand, and on the other hand, decreases social functioning by discouraging patients from seeking help from family and society. Conversely, the differences in physical functioning, role functioning, and cognitive functioning are more significantly affected by treatment. Yue Li et al. once conducted a meta-analysis to compare patients’ HRQoL status between breast conservation treatment (BCT) and mastectomy, with the results showing significant differences in the levels of the physical, role, and cognitive functioning of patients who received different treatments (65). Moreover, Eman et al. (66) also demonstrated that hormonotherapy could maintain a better functional status for patients than could chemotherapy and radiotherapy.

Although sexual enjoyment presented the lowest score in our study, the score of sexual functioning was similar to that of sexual enjoyment, and both had a negative status, which is different from the studies conducted in Latin America and the Caribbean (67), where patients had different scores for sexual enjoyment and sexual functioning. The decline of the sexual functioning of BC patients could be caused by various treatments, such as the commonly used drugs tamoxifen and aromatase inhibitors in adjuvant endocrine therapy, whose side effects of vaginal dryness and dyspareunia are often reported in reviews (68). Research has also demonstrated that for cancer patients, conserving the breast or not has a significant impact on their sexual wellbeing and satisfaction since the breast is regarded as a secondary sexual characteristic (69). In addition, patients from different cultural backgrounds have quite different attitudes toward sexual knowledge and sexual life. Compared to Western patients, Asian patients treat their sexual life as a private topic and do not like to discuss it publicly; moreover, they even choose random answers when responding to questionnaires to avoid exposing their privacy (32, 40). Thus, healthcare professionals should consider the possibility of inconsistency between the outcomes evaluated and the actual HRQoL status when they provide medical and nursing care for Asian BC patients.

With respect to the assessment of body image, our study’s results are almost indistinguishable from those of Polish scholars (70): BC patients’ perception of their body image decreases after surgery. Montazeri et al. and Arora et al. stated that a deterioration in women’s perception of their body may worsen, even after their general physical condition has recovered and symptoms have been alleviated after surgery and subsequent systemic treatments (71, 72). Influenced by the negative emotion of anxiety, patients are usually pessimistic about their future, body image, and sexual functioning (73). Agnieszka showed that a higher level of emotional, cognitive, and social functioning cannot only help prevent negative assessment of scars in women but can also ensure a better perception of both their body image and future prospects (70). Therefore, paying long-term attention to the BC patients’ functional status and providing them with dynamic interventions according to updated psychological data are essential to integrally improving and maintaining their quality of life.

### The experience of symptoms of Asian breast cancer patients

Along with a decline in functioning comes a battery of symptoms caused by drugs, surgeries, and other treatments that damage BC patients’ quality of life. Fatigue and being upset by hair loss are the common symptoms of BC patients in Asia. Cancer-related fatigue (CRF)—a persistent state of severe exhaustion—impairs BC patients’ quality of life. It not only makes patients’ functioning abnormal but also gives rise to an increase in BC incidence and mortality. Nevertheless, CRF is underestimated and underreported by physicians and patients (74–76). Currently, the pathophysiological mechanism of CRF remains unclear, except that surgery, chemotherapy, and hormonotherapy are associated with CRF. Also potentially contributing to different cancer outcomes are the following: less or no access to quality care, differences in tumor biology, and socioeconomic factors influencing treatment options that may be affected by racial differences (77, 78). These may help explain why, when we compared our results to those of Lucas, the degree of fatigue of patients in Asia is more severe than those in Latin America and the Caribbean (67). The etiology and pathogenesis of fatigue in BC patients are complicated and multicausal. Many studies have demonstrated that CRF and other symptom clusters, such as sleep disturbance, mood disorder, and pain, are simultaneous (79). The results of these studies are consistent with our conclusion that fatigue, pain, and insomnia affect BC patients’ quality of life to a similar extent. Moreover, inflammation may be the key biological mechanism underlying this symptom cluster since a prior study found that the coexistence of arthralgia, fatigue, and insomnia was associated with an increased level of inflammatory biomarkers among women on endocrine therapy (80).

As evaluated by the symptom scale of EORTC QLQ-C30, financial difficulty is also a serious problem that negatively impacts Asian patients’ quality of life. In comparison, Asian BC patients experience greater financial pressure than their American counterparts (67) but less than Egyptians (66). In addition to age, gender, marital status, monthly net income, educational level, and self-reported health status, national income level and health insurance coverage are also associated with financial hardship among BC patients (81–85), which suggests that with all relevant factors taken into consideration, designing a matching benefit package is essential to reducing the financial burden on patients.

Being upset by hair loss is another serious symptom of Asian BC patients according to this meta-analysis. Hair loss is one of the distressing side effects for BC patients who undergo chemotherapy. Hence, we here allude to the term chemotherapy-induced alopecia (CIA), which is usually an unavoidable but transient side effect that can be dealt with by wearing wigs. BC patients are mostly women, and they regard hair as an integral part of their identity. CIA, however, affects their social life, bringing them physical and psychological distress. More seriously, some BC patients may bear the agony of hair loss even after 6 months of chemotherapy, leading to low self-esteem and lower HRQoL (86). Chemotherapy-induced irreversible alopecia (CIIA) usually occurs after high-dose chemotherapy, and married women seem to be more upset about hair loss than others (86, 87). Therefore, some preventive measures, such as scalp cooling or psychological intervention, should be implemented for high-risk CIIA patients to prevent or reduce the degree of their hair loss and emotional distress. Nausea and vomiting, dyspnea, loss of appetite, constipation, and breast and arm symptoms may still affect patients’ quality of life. However, the impact is much lighter than those symptoms mentioned above, perhaps because these symptoms usually appear during the acute phase of treatments and can be controlled by specific traditional medicines in Asia (88, 89).

This review used two tools to evaluate the HRQoL of BC patients, namely, EORTC QLQ-C30 and QLQ-BR23. Both tools represent patient-reported outcomes (PRO), which demonstrate the patients’ physical, psychological, and social response to disease and therapy from their own perspective but not from that of the physician or anyone else. The evaluation clearly shows that the patients’ functioning and symptoms interact with each other, jointly leading to the decline of HRQoL in BC patients. We therefore suggest that a holistic assessment of BC patients is necessary to provide targeted psychological and medical treatments and enhance their quality of life.

Nevertheless, there are still some limitations to this article. First, likely due to the specific methodological limitations of cross-sectional studies included in the meta-analysis, considerable heterogeneity was observed among the studies that evaluated the HRQoL of BC patients. Second, only studies published in English and Chinese were searched, and research projects that did not provide merged data were excluded. Third, gray literature was excluded from our review because of the difficulty of conducting a quality assessment without a detailed description of the methodology and peer review.

## Conclusion

BC patients in Asia have a lower HRQoL under the high prevalence, growing incidence, and advanced breast cancer diagnosis at an earlier age. As assessed by EORTC QLQ-C30 and QLQ-BR23, Asian BC patients have different degrees of impairment in physical, social, sexual, and other functioning and suffer from various symptoms, including, but not limited to, fatigue, pain, insomnia, and the influence of financial difficulties, resulting to decline in their quality of life. Therefore, raising awareness of routine breast cancer screening in the Asian population is the first fundamental measure to curb BC deterioration and avoid s adverse impact on HRQoL. Since the symptoms of BC affect the body functions of BC patients, and serious symptoms of this illness can lead to psychological dilemma and mental disorder and vice versa, the mutual effects change over time; thus, and a dynamic assessment of BC patients and a corresponding treatment plan are essential and should be ensured. Psychological interventions with one-on-one psychotherapy and cognitive behavioral therapy (CBT) can also be introduced to relieve patients’ negative emotions caused by poor body image perceptions, such as embarrassment about hair loss and uneasiness about visible scarring. In addition, encouraging patients to seek help from their social networks could be an important approach to improving their psychological state. For pain, insomnia, arm dysfunction, and other symptoms, it is recommended that caregivers use traditional Asian medicine to help BC patients alleviate those symptoms in a cost-effective way. Finally, in view of the high prevalence of breast cancer in Asia, each country in Asia should do its best to increase the coverage of their healthcare systems so that BC patients’ financial difficulties may be alleviated.

## Data availability statement

The original contributions presented in the study are included in the article and **Supplementary Material**. Further inquiries can be directed to the corresponding authors.

## Author contributions

All authors contributed to the article and approved the version submitted. JG and CH proposed the concept and monitored the progress of the work. CH provided the ideas for article revisions. CW and DB designed the work and provided solutions to the inconsistencies. XC, CW, and TC performed the literature search, study selection, and data extraction, and then wrote the manuscript. XC and CW undertook data analysis and interpretation. XC, CW, and DB wrote the manuscript and made the equal contributions to the article. Meanwhile, LZ and HL helped to review and check the manuscript. CW and DB contributed to the revision of the manuscript. All the authors approved the submitted version and agreed to be personally accountable to their own contributions and to ensure that questions related to the accuracy or integrity of any part of the work are addressed.

## Funding

This study was supported by grants from the Sichuan Federation of Social Science Associations (NO. SC22B149 and NO. SC22B150), the Health Commission of Sichuan Province (NO. 21PJ109), and the Sichuan Mental Health Education Research Center (NO. XLJKJY2203A).

## Conflict of interest

The authors declare that the research was conducted in the absence of any commercial or financial relationships that could be construed as a potential conflict of interest.

## Publisher’s note

All claims expressed in this article are solely those of the authors and do not necessarily represent those of their affiliated organizations, or those of the publisher, the editors and the reviewers. Any product that may be evaluated in this article, or claim that may be made by its manufacturer, is not guaranteed or endorsed by the publisher.
