# Supplementary material for: Health-related quality of life in breast cancer patients in Asia: A meta-analysis and systematic review
Source: Front Oncol. 2022 Sep 28;12:954179. doi: 10.3389/fonc.2022.954179 (PMC9554636; doi:10.3389/fonc.2022.954179)
Supplement: Supplementary file 1 [file DataSheet_1.doc]

**Supplementary Materials**

**Supplementary materials caption**

Appendix S1 Literature search strategies

Appendix S2 Quality appraisal of the studies

Appendix S3 Assessment results for the risk of bias

**Appendix S1 Literature search strategies**

**Pubmed**

#1 (“Asia, Southeastern” OR “Far East” OR “Southeast Asia” OR “South eastern Asia” OR “South central” OR “Asia, Western” OR “Middle East” OR China OR Hong Kong OR Macau OR Taiwan OR Tibet OR Japan OR Korea OR Mongolia OR India OR Brunei OR Indonesia OR Malaysia OR Myanmar OR Singapore OR Thailand OR Timor-Leste OR Vietnam OR Bangladesh OR Bhutan OR Nepal OR Pakistan OR “Sri Lanka” OR Kazakhstan OR Tajikistan OR Turkmenistan OR Borneo)[Mesh] OR (“Asia, Southeastern” OR “Far East” OR “Southeast Asia” OR “South eastern Asia” OR “South central” OR “Asia, Western” OR “Middle East” OR China OR Chine* OR Hong Kong* OR Macau OR Tibet* OR Taiwan* OR Japan* OR Korea* OR Mongoli* OR India* OR Brunei* OR Indonesia* OR Lao* OR Malay* OR Myanmar OR Burmese* OR Philippin* OR Singapore* OR Thai* OR Timor* OR Vietnam* OR Bangladesh OR Bengal* OR Bhutan* OR India* OR Nepal* OR Pakistan* OR “Sri Lanka*” OR Kazakhstan OR Tajikistan OR Turkmenistan OR Borneo )[Title/Abstract]

#2 "breast cancer"[Title/Abstract] OR "breast neoplasm"[Title/Abstract] OR "breast tumor"[Title/Abstract] OR "mammary cancer"[Title/Abstract] OR "mammary neoplasm"[Title/Abstract] OR "mammary tumor"[Title/Abstract] OR "breast neoplasms"[MeSH Terms]

#3 "quality of life"[Title/Abstract] OR "health-related quality of life"[Title/Abstract] OR "health-related quality of life"[Title/Abstract] OR "QoL"[Title/Abstract] "HRQoL"[Title/Abstract] OR "quality of life"[MeSH Terms]

#4 #1 and #2 and #3

**Web of science**

#1 (“Asia, Southeastern” OR “Far East” OR “Southeast Asia” OR “South eastern Asia” OR “South central” OR “Asia, Western” OR “Middle East” OR China OR Chine* OR Hong Kong* OR Macau OR Tibet* OR Taiwan* OR Japan* OR Korea* OR Mongoli* OR India* OR Brunei* OR Indonesia* OR Lao* OR Malay* OR Myanmar OR Burmese* OR Philippin* OR Singapore* OR Thai* OR Timor* OR Vietnam* OR Bangladesh OR Bengal* OR Bhutan* OR India* OR Nepal* OR Pakistan* OR “Sri Lanka*” OR Kazakhstan OR Tajikistan OR Turkmenistan OR Borneo )[TI,TS,AB)

#2 (“breast cancer” OR “breast neoplasm” OR “breast tumor” OR “mammary cancer” OR “mammary neoplasm” OR “mammary tumor”)[TI,TS,AB]

#3 (“quality of life” OR “health-related quality of life” OR “health related quality of life” OR QoL OR HRQoL)[TI,TS,AB)

#4 #1 and #2 and #3

**Embase**

#1 (“Asia, Southeastern” OR “Far East” OR “Southeast Asia” OR “South eastern Asia” OR “South central” OR “Asia, Western” OR “Middle East” OR China OR Chine* OR Hong Kong* OR Macau OR Tibet* OR Taiwan* OR Japan* OR Korea* OR Mongoli* OR India* OR Brunei* OR Indonesia* OR Lao* OR Malay* OR Myanmar OR Burmese* OR Philippin* OR Singapore* OR Thai* OR Timor* OR Vietnam* OR Bangladesh OR Bengal* OR Bhutan* OR India* OR Nepal* OR Pakistan* OR “Sri Lanka*” OR Kazakhstan OR Tajikistan OR Turkmenistan OR Borneo )[TI,AB)

#2 (“breast cancer” OR “breast neoplasm” OR “breast tumor” OR “mammary cancer” OR “mammary neoplasm” OR “mammary tumor”)[TI,AB]

#3 (“quality of life” OR “health-related quality of life” OR “health related quality of life” OR QoL OR HRQoL )[TI,AB)

#4 #1 and #2 and #3

**Cochrane**

#1 (“Asia, Southeastern” OR “Far East” OR “Southeast Asia” OR “South eastern Asia” OR “South central” OR “Asia, Western” OR “Middle East” OR China OR Chine* OR Hong Kong* OR Macau OR Tibet* OR Taiwan* OR Japan* OR Korea* OR Mongoli* OR India* OR Brunei* OR Indonesia* OR Lao* OR Malay* OR Myanmar OR Burmese* OR Philippin* OR Singapore* OR Thai* OR Timor* OR Vietnam* OR Bangladesh OR Bengal* OR Bhutan* OR India* OR Nepal* OR Pakistan* OR “Sri Lanka*” OR Kazakhstan OR Tajikistan OR Turkmenistan OR Borneo )[TKA)

#2 (“breast cancer” OR “breast neoplasm” OR “breast tumor” OR “mammary cancer” OR “mammary neoplasm” OR “mammary tumor”)[TKA]

#3 (“quality of life” OR “health-related quality of life” OR “health related quality of life” OR QoL OR HRQoL )[TKA)

#4 #1 and #2 and #3

**Psyclnfo**

#1 (“Asia, Southeastern” OR “Far East” OR “Southeast Asia” OR “South eastern Asia” OR “South central” OR “Asia, Western” OR “Middle East” OR China OR Chine* OR Hong Kong* OR Macau OR Tibet* OR Taiwan* OR Japan* OR Korea* OR Mongoli* OR India* OR Brunei* OR Indonesia* OR Lao* OR Malay* OR Myanmar OR Burmese* OR Philippin* OR Singapore* OR Thai* OR Timor* OR Vietnam* OR Bangladesh OR Bengal* OR Bhutan* OR India* OR Nepal* OR Pakistan* OR “Sri Lanka*” OR Kazakhstan OR Tajikistan OR Turkmenistan OR Borneo )[ab,mh,ti]

#2 (“breast cancer” OR “breast neoplasm” OR “breast tumor” OR “mammary cancer” OR “mammary neoplasm” OR “mammary tumor”)[ab,mh,ti]

#3 (“quality of life” OR “health-related quality of life” OR “health related quality of life” OR QoL OR HRQoL )[ab,mh,ti]

#4 #1 and #2 and #3

**CINAHL**

#1 (“Asia, Southeastern” OR “Far East” OR “Southeast Asia” OR “South eastern Asia” OR “South central” OR “Asia, Western” OR “Middle East” OR China OR Chine* OR Hong Kong* OR Macau OR Tibet* OR Taiwan* OR Japan* OR Korea* OR Mongoli* OR India* OR Brunei* OR Indonesia* OR Lao* OR Malay* OR Myanmar OR Burmese* OR Philippin* OR Singapore* OR Thai* OR Timor* OR Vietnam* OR Bangladesh OR Bengal* OR Bhutan* OR India* OR Nepal* OR Pakistan* OR “Sri Lanka*” OR Kazakhstan OR Tajikistan OR Turkmenistan OR Borneo )[ab,su,ti]

#2 (“breast cancer” OR “breast neoplasm” OR “breast tumor” OR “mammary cancer” OR “mammary neoplasm” OR “mammary tumor”)[ab,su,ti]

#3 (“quality of life” OR “health-related quality of life” OR “health related quality of life” OR QoL OR HRQoL)[ab,su,ti]

#4 #1 and #2 and #3

CNKI

#1 (乳腺癌 + “breast cancer” + “breast neoplasm” + “breast tumor” + “mammary cancer” + “mammary neoplasm” + “mammary tumor”)[su]

#2 (生活质量 + 健康相关生活质量 + “quality of life” + “health-related quality of life” + “health related quality of life” + QoL + HRQoL)

#3 #1 and #2

**Appendix S2 Quality appraisal of the studies**

| Study ID,Year | ① | ② | ③ | ④ | ⑤ | ⑥ | ⑦ | ⑧ | Overall appraisal |
| --- | --- | --- | --- | --- | --- | --- | --- | --- | --- |
| Dubashi B 2010 | Yes | Yes | Yes | Yes | No | No | Yes | Yes | High |
| Ghufran 2013 | Yes | Yes | Yes | Yes | Yes | Yes | Yes | Yes | High |
| Min2020 | Yes | Yes | Yes | Yes | No | No | Yes | Yes | High |
| Qing Chen2018 | Yes | Yes | Yes | Yes | Yes | Yes | Yes | Yes | High |
| Muna 2018 | Yes | Yes | Yes | Yes | Yes | Yes | Yes | Yes | High |
| Huang2019 | Yes | Yes | Yes | Yes | Yes | Yes | Yes | Yes | High |
| F. Najaf2016 | Yes | Yes | Yes | Yes | No | No | Yes | Yes | High |
| Fatemeh 2021 | Yes | Yes | Yes | Yes | No | No | Yes | Yes | High |
| Safaee A2008 | Yes | Yes | Yes | Yes | No | No | Yes | Yes | High |
| K.M. Almutairi2016 | Yes | Yes | Yes | Yes | Yes | Yes | Yes | Yes | High |
| Najmeh 2013 | Yes | Unclear | Yes | Yes | No | No | Yes | Yes | High |
| Aishwarya 2019 | Yes | Unclear | Yes | Yes | No | No | Yes | Yes | High |
| Sri Ganesh2016 | Yes | Yes | Yes | Yes | Yes | Yes | Yes | Yes | High |
| Sajani 2014 | Yes | Unclear | Yes | Yes | No | No | Yes | Yes | High |
| Azlina 2013 | Yes | Yes | Yes | Yes | No | No | Yes | Yes | High |
| Ahmet 2009 | Yes | Yes | Yes | Yes | No | No | Yes | Yes | High |
| Huang2017 | Yes | Yes | Yes | Yes | Yes | Yes | Yes | Yes | High |
| Syarifah 2022 | Yes | Yes | Yes | Yes | Yes | Yes | Yes | Yes | High |
| Huda 2012 | Yes | Yes | Yes | Yes | Yes | Yes | Yes | Yes | High |
| Shafika 2009 | Yes | Yes | Yes | Yes | Yes | Yes | Yes | Yes | High |
| Fatemeh 2017 | Yes | Yes | Yes | Yes | Yes | Yes | Yes | Yes | High |
| Saleha S B2010 | Yes | Yes | Yes | Yes | Yes | Yes | Yes | Yes | High |
| Fahimeh 2018 | Yes | Yes | Yes | Yes | Unclear | Unclear | Yes | Yes | High |
| ①Were the criteria for inclusion in the sample clearly defined?；②Were the study subjects and the setting described in detail?；③Was the exposure measured in a valid and reliable way?；④Were objective, standard criteria used for measurement of the condition?；⑤Were confounding factors identified?；⑥Were strategies to deal with confounding factors stated?；⑦Were strategies to deal with confounding factors stated?；⑧Was appropriate statistical analysis used? | | | | | | | | | |

| **Study ID,Year** | **①** | **②** | **③** | **④** | **⑤** | **⑥** | **⑦** | **Overall bias** |
| --- | --- | --- | --- | --- | --- | --- | --- | --- |
| Dubashi B 2010 | Moderate | Moderate | Moderate | Moderate | Serious | Moderate | Serious | Serious |
| Ghufran 2013 | Low | Low | Low | Low | Low | Moderate | Low | Low |
| Min 2020 | Moderate | Moderate | Low | Moderate | Low | Moderate | Low | Moderate |
| Qing Chen2018 | Moderate | Moderate | Moderate | Moderate | Low | Moderate | Low | Moderate |
| Muna 2018 | Moderate | Moderate | Moderate | Moderate | Moderate | Moderate | Low | Moderate |
| Huang2019 | Low | Low | Low | Low | Low | Moderate | Low | Low |
| F. Najaf2016 | Moderate | Moderate | Moderate | Moderate | Low | Moderate | Low | Moderate |
| Fatemeh 2021 | Moderate | Moderate | Moderate | Moderate | Low | Moderate | Low | Moderate |
| Safaee A2008 | Moderate | Moderate | Moderate | Moderate | Serious | Moderate | Serious | Serious |
| K.M. Almutairi2016 | Moderate | Moderate | Moderate | Moderate | Low | Moderate | Low | Moderate |
| Najmeh 2013 | Moderate | Moderate | Moderate | Moderate | Low | Moderate | Low | Moderate |
| Aishwarya2019 | Moderate | Moderate | Moderate | Moderate | Serious | Moderate | Serious | Serious |
| Sri Ganesh2016 | Moderate | Moderate | Moderate | Moderate | Low | Moderate | Low | Moderate |
| Sajani 2014 | Moderate | Moderate | Moderate | Moderate | Low | Moderate | Low | Moderate |
| Azlina2013 | Low | Low | Moderate | Moderate | Serious | Moderate | Serious | Serious |
| Ahmet 2009 | Moderate | Moderate | Moderate | Moderate | Low | Moderate | Moderate | Moderate |
| Huang2017 | Low | Low | Low | Low | Low | Moderate | Low | Low |
| Syarifah 2022 | Low | Low | Low | Low | Low | Moderate | Low | Low |
| Huda 2012 | Moderate | Moderate | Moderate | Moderate | Low | Moderate | Low | Moderate |
| Shafika 2009 | Moderate | Moderate | Moderate | Moderate | Low | Moderate | Low | Moderate |
| Fatemeh 2017 | Moderate | Moderate | Moderate | Moderate | Serious | Moderate | Serious | Serious |
| Saleha 2010 | Moderate | Moderate | Moderate | Moderate | Low | Moderate | Low | Moderate |
| Fahimeh 2018 | Moderate | Moderate | Moderate | Moderate | Low | Moderate | Low | Moderate |
| ①Bias due to confounding;  ②Bias in selection of participants into the study;  ③Bias in classification of interventions;  ④Bias due to deviations from intended interventions;  ⑤Bias due to missing data;  ⑥Bias in measurement of outcomes；  ⑦Bias in selection of the reported result.  Low risk: comparable to a well-performed randomized trial. Moderate risk: sound for a non-randomized study but cannot be considered comparable to a well-performed randomized trial. Serious risk: some important problems in this domain. Critical risk: too problematic in this domain to provide any useful evidence on the effects of intervention. No information (NI) to base a judgment on risk of bias for this domain | | | | | | | | |

**Appendix S3 Assessment results for the risk of bias**
